# Study of the Plasma and Buffy Coat in Patients with SARS-CoV-2 Infection—A Preliminary Report

**DOI:** 10.3390/pathogens10070805

**Published:** 2021-06-25

**Authors:** Karla B. Peña, Francesc Riu, Josep Gumà, Carmen Guilarte, Berta Pique, Anna Hernandez, Alba Àvila, Sandra Parra, Antoni Castro, Conxita Rovira, Pitter Cueto, Immaculada Vallverdu, David Parada

**Affiliations:** 1 Pathology Molecular Unit, Department of Pathology, Hospital Universitari de Sant Joan, Institut d’Investigació Sanitària Pere Virgili, Facultat de Medicina, Universitat Rovira i Virgili, 43204 Reus, Spain; karlabeatriz.pena@grupsagessa.com (K.B.P.); friu@grupsagessa.com (F.R.); cguilarte@grupsagessa.com (C.G.); berta.pique@gmail.com (B.P.); anna.hernandeza@gmail.com (A.H.); 2 Department of Oncology, Hospital Universitari de Sant Joan, Institut d’Investigació Sanitària Pere Virgili, Facultat de Medicina, Universitat Rovira i Virgili, 43204 Reus, Spain; 3 Department of Internal Medicine, Hospital Universitari de Sant Joan, Institut d’Investigació Sanitària Pere Virgili, Facultat de Medicina, Universitat Rovira i Virgili, 43204 Reus, Spain; alba.avila@iisp.com (A.À.); sandra.parra@urv.cat (S.P.); antoni.castro@urv.cat (A.C.); 4 Department of Intensive Care Medicine, Hospital Universitari de Sant Joan, Institut d’Investigació Sanitària Pere Virgili, Facultat de Medicina, Universitat Rovira i Virgili, 43204 Reus, Spain; croviraangels@gmail.com (C.R.); pittercueto@gmail.com (P.C.); ivallverdu@grupsagessa.com (I.V.)

**Keywords:** SARS-CoV-2, RNA, plasma, buffy coat, blood, immunohistochemical, morphology

## Abstract

The pandemic caused by the SARS-CoV-2 infection affects many aspects of public health knowledge, science, and practice around the world. Several studies have shown that SARS-CoV-2 RNA in plasma seems to be associated with a worse prognosis of COVID-19. In the present study, we investigated plasma and buffy RNA in patients with COVID-19 to determine its prognostic value. A prospective study was carried out in patients hospitalized for COVID-19, in which RNA was analyzed in plasma and the buffy coat. Morphological and immunohistochemical studies were used to detect the presence of SARS-CoV-2 in the buffy coat. In COVID-19 patients, the obtained RNA concentration in plasma was 448.3 ± 31.30 ng/mL. Of all the patients with positive plasma tests for SARS-CoV-2, 46.15% died from COVID-19. In four cases, tests revealed that SARS-CoV-2 was present in the buffy coat. Abnormal morphology of monocytes, lymphocytes and neutrophils was found. An immunohistochemical study showed positivity in mononuclear cells and platelets. Our results suggest that SARS-CoV-2 is present in the plasma. This facilitates viral dissemination and migration to specific organs, where SARS-CoV-2 infects target cells by binding to their receptors. In our study, the presence of plasma SARS-CoV-2 RNA was correlated with worse prognoses.

## 1. Introduction

SARS-CoV-2 is the causative agent of COVID-19, an enveloped positive-sense single-stranded RNA virus that belongs to a large family of coronaviruses [1,2,3,4]. The SARS-CoV-2 genome consists of 14 open reading frames (ORFs), 9 of which encode 16 non-structural proteins (nsp1–16) [4,5]. The remaining five frames encode nine accessory proteins (ORFs) and four structural proteins: spike (S), envelope (E), membrane (M), and nucleocapsid (N) [4,5]. As is well known, COVID-19 has significantly and continuously increased the number of hospitalizations alongside the development of concomitant multi-organ diseases [1,2,3,4].

SARS-CoV-2 infection can be asymptomatic, or it can cause a wide spectrum of symptoms, such as mild upper respiratory infection and life-threatening sepsis. However, the presence of SARS-CoV-2 in peripheral blood remains a matter of debate due to the implications it has during the disease and its correlation with prognostic and predictive factors. In the present study, we investigated the presence of SARS-CoV-2 in both plasma and the buffy coat in order to understand the way in which the hematogenous spread of the virus occurs and explain the mechanisms of systemic viral involvement.

## 2. Results

### 2.1. Clinical Findings

Our sample consisted of 18 male and 6 female patients with a median age of 66.80 years (range, 51–82 years). Systemic arterial hypertension (58.33%), dyslipidemia (54.67%), type 2 diabetes mellitus (25%), and obesity (12.5%) were the predominant comorbidities in the sample. Two patients had a history of colonic and breast carcinoma. Two (8.33%) patients were asymptomatic, and twenty-two patients (91.67%) showed symptoms of SARS-CoV-2 infection, such as fever, dry cough, shortness of breath, fatigue, rhinorrhea, respiratory distress, and diarrhea. Twenty (83.33%) patients developed pneumonia, of whom thirteen required admission to the intensive care unit. The mean hospitalization time was 35.76 days (range, 2–91). Eight patients (33.33%) died from COVID-19. Treatment was individualized in each patient and consisted of a single or combined treatment of high-flow oxygen therapy, dexamethasone, remdesivir, antibiotic therapy (ceftriaxone–azithromycin), or anticoagulants. Eighteen patients received dexamethasone, and three patients met the clinical criteria for remdesivir. Table 1 and Table 2 summarize the results.

### 2.2. RNA Plasma Findings

**Figure 1:**
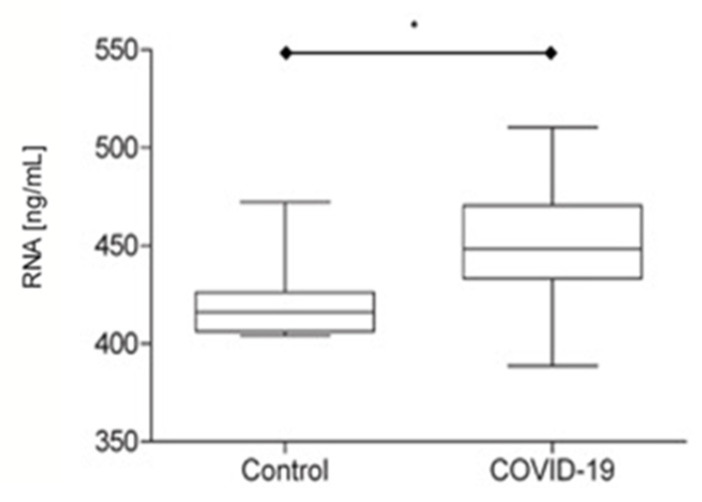
Plasma RNA concentration in the control group (N = 8) and COVID-19 patients (N = 24) (* *p* < 0.05).

**Figure 2:**
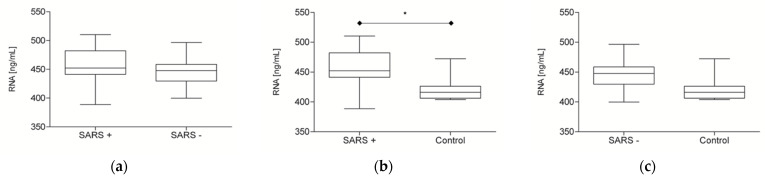
Plasma RNA concentration in SARS-CoV-2-positive (N = 13), SARS-CoV-2-negative (N = 11) and control groups (N = 8). (**a**) SARS-positive group vs. SARS-negative group (*p* ≥ 0.05). (**b**) SARS-positive group vs. control group (* *p* < 0.05). (**c**) SARS-negative group vs. control group (*p* ≥ 0.05).

In the twenty-four COVID-19 patients, the RNA plasma concentration obtained was 448.3 ± 31.30 ng/mL, and in the control group, the plasma RNA concentration was 416.2 ± 22.24 ng/mL. A significant difference was observed between the groups (*p* < 0.05) (Figure 1). Eleven patients (45.83%) returned a negative RT-PCR SARS-CoV-2 test (447.8 ± 28.56 ng/mL plasma RNA concentration), and thirteen (54.17%) showed a presence of viral genetic components (452.2 ± 33.84 ng/mL plasma RNA concentration). In COVID-19 patients, no significant differences were observed between plasma RT-PCR SARS-CoV-2 positive and plasma RT-PCR SARS-CoV-2 negative (Figure 2a). When SARS-CoV-2-positive and control groups were compared, significant differences were observed (Figure 2b). No differences were observed between SARS-CoV-2-negative and control groups (Figure 2c). Six patients (46.15%) with positive plasma RT-PCR tests for SARS-CoV-2 died from COVID-19 complications. Two patients (18.18%) with negative plasma RT-PCR tests for SARS-CoV-2 died due to complications from COVID-19. No significant differences in plasma RNA concentration were found between those patients who lived and died.

**Figure 3:**
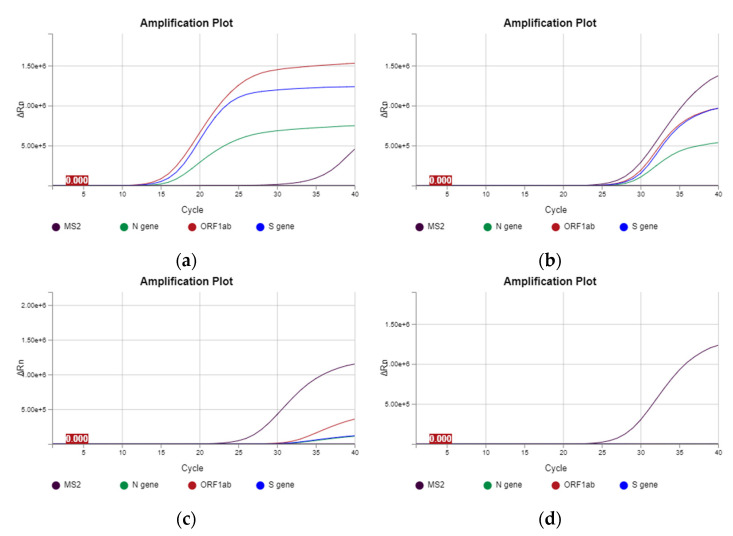
Plasma amplification plot of patients with SARS-CoV-2. (**a**,**b**) Amplification plot of SARS-CoV-2-positive patients with gene expressions of N, ORF and S. (**c**) Amplification plot of SARS-CoV-2-positive patient with gene expressions of ORF and S. (**d**) Amplification plot of a SARS-CoV-2-negative patient, showing the internal positive control MS2 and negative control (flat line).

**Figure 4:**
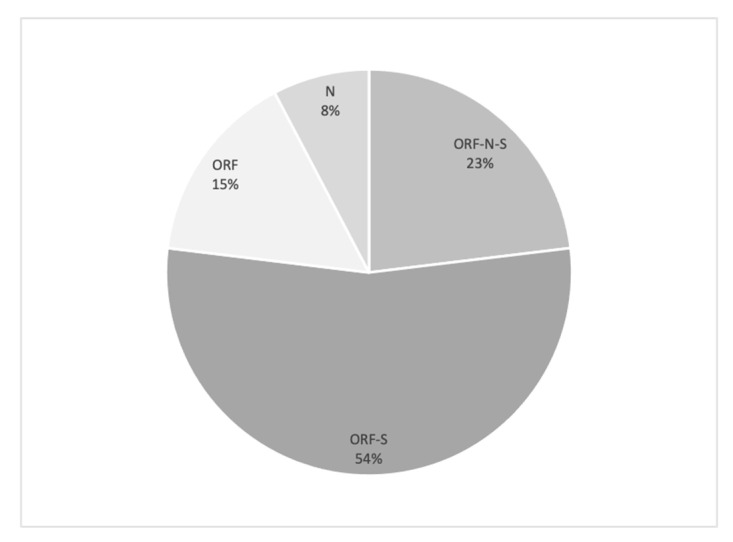
Gene distribution of plasma concentration in SARS-CoV-2-positive patients (N = 13).

Three patients (23.08%) showed ORF, N and S gene positivity (Figure 3a,b). ORF and S gene positivity was observed in seven patients (53.85%). The ORF gene was present in two patients (15.38%) and the N gene in one (7.29%) (Figure 3c). The mean SARS-CoV-2 load was 41.106 copies/mL (range, 37.710–44.597 copies/mL). Table 3 and Figure 4 summarize the findings.

### 2.3. RNA Buffy Coat Findings

The RNA concentration in the buffy coat was 18.15 ± 23.53 ng/mL. In four patients (16.67%), the RT-PCR SARS-CoV-2 test showed the presence of viral genetic components. ORF and N genes were present in two patients (50%), N and S genes in one patient (25%), and the N gene in one patient (25%). In the four patients with genetic viral components in the buffy coat, the plasma RNA gene was also present, but less or equal genes were detected. Table 3 summarizes the results.

### 2.4. Morphological and Immunohistochemical Buffy Coat Findings

#### 2.4.1. Morphological Study

**Figure 5:**
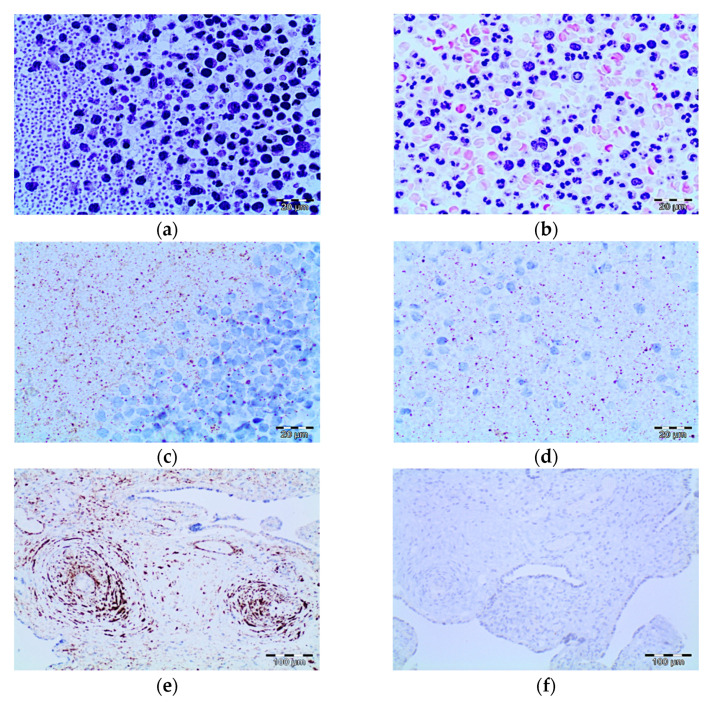
Morphological and immunohistochemistry findings in the buffy coat in patients positive for SARS-CoV-2 shown by RT-PCR. (**a**,**b**) The images show neutrophils with nuclear hyposegmentation and hypergranular cytoplasm. Mononuclear cells also showed atypical findings with a nucleus/ratio increase and apoptotic bodies (HE, 20×). (**c**,**d**) Immunohistochemistry study showing granular cytoplasm positive reaction in mononuclear cells and platelets (DAB, 20×). (**e**,**f**) Positive and negative control (DAB, 10×).

The morphological study of the buffy coat revealed morphological variations in the components of both the white cells and platelets. In polymorphonuclear cells, cytoplasmic granulation increased; nuclear segmentation decreased; and occasional inclusions, similar to Howell–Jolly bodies, were observed (Figure 5a,b). Furthermore, larger lymphocytes with plasmacytoid characteristics were observed. Occasional atypical monocytes with cytoplasmic vacuolization were also observed (Figure 5a). In platelets, we observed an increase in size focally, with occasional irregularities in the cytoplasmic membrane (Figure 5b).

#### 2.4.2. Immunohistochemical Study

We investigated two types of antibodies to SARS-CoV-2 that were standardized in our laboratory and that immunoreact with protein S and the nucleoprotein (NP). For the two antibodies investigated, we observed granular cytoplasmic reactivity of varying intensity in occasional lymphocytes, monocytes and platelets. This immunoreactivity was observed in all cases with a positive RT-PCR result for SARS-CoV-2. Three cases with a negative RT-PCR result for SARS-CoV-2 also showed this type of cellular expression pattern (Figure 5c,d). The positive control (placenta from a patient with SARS-CoV-2 infection) showed granular cytoplasmic immunoreactivity in smooth muscle and endothelial cells. Granular reactivity was also observed in isolated stromal cells. In the negative control, no immunoreaction was found for either of the two antibodies investigated (Figure 5e).

## 3. Discussion

In the present study, we analyzed a relatively small sample of hospitalized COVID-19 patients. Nonetheless, we found findings comparable with those of other studies in relation to gender, mortality, etc. In our study, we found SARS-CoV-2 plasma RNA in thirteen COVID-19 patients (54.17%) who required care in the intensive care unit, of whom six (46.15%) died from COVID-19. In contrast, two of the eleven patients (18.18%) with no SARS-CoV-2 plasma RNA died from the infection. These findings are supported by those of previous studies, in which SARS-CoV-2 plasma RNA was associated with a worse clinical prognosis in patients with COVID-19 [6,7,8,9,10,11,12,13,14,15,16]. In addition, the quantification of viral RNA showed that COVID-19 patients generally had higher amounts of plasma RNA than those of the control group (lung cancer patients at follow-up). This finding may be related to the presence of viral RNA in plasma since the COVID-19 patients that were positive for SARS-CoV-2 showed a significant difference compared to that of the control group, while the COVID-19 patients with no plasma viral RNA did not. However, some studies have reported that the presence of plasma viral RNA may be related to the post-viral state [16,17], the cytokine storm or the idiosyncrasy of the patient [16,17] rather than to the active infection. Our study suggests that the absolute quantification of plasma RNA in patients with COVID-19, regardless of its origin, could serve as a variable to examine the clinical evolution of patients. Furthermore, the presence of SARS-CoV-2 RNA in plasma, together with other clinical factors, such as age, obesity, diabetes and arterial hypertension, could be associated with a worse prognosis in individuals with COVID-19. In our study, we demonstrated the presence of viral components in 13 out of 24 patients (54.17%) by plasma RT-PCR. However, RT-qPCR has been reported to exhibit poor and highly variable diagnostic sensitivity (1–40%) when employed to detect SARS-CoV-2 RNA in blood samples from confirmed COVID-19 cases, with most positive samples exhibiting high Ct values indicative of a low viral RNA concentration [18]. More research is needed to shed light on the prognostic significance of plasma viral RNA in COVID-19 patients and its relationship with predictive factors.

The buffy coat analysis showed lower concentrations of RNA in patients with COVID-19 than in plasma from the same patients. We demonstrated the presence of genetic material of SARS-CoV-2 in the buffy coat in only 4 patients, whereas plasma viral RNA was present in 13. Likewise, we found that more SARS-CoV-2-specific genes were expressed in plasma than in the buffy coat. This finding suggests that the presence of SARS-CoV-2 genes at the buffy coat level is a consequence of viral RNA fragmentation or the degenerative stages of the virus. Another possible explanation is that the virions are shed during processing due to labile unions with the various cellular elements. However, the buffy coat study is less useful for determining viral RNA and suggests that SARS-CoV-2 is predominantly found in free form in the blood. The free hematogenous spread of SARS-CoV-2 can explain its systemic involvement in COVID-19. Furthermore, its infective capacity is related to target organs capable of expressing ACE2 and TMPRSS2 receptors, allowing their cellular internalization and viral replication. However, some research has shown the limited replicative capacity of SARS-CoV-2 in peripheral blood monocytes and macrophages, and this finding could be due to a higher concentration of viral RNA in plasma, as found in our study [19,20]. Additional studies need to be conducted on the dynamics of SARS-CoV-2 infection in peripheral blood.

The findings of the morphological study of the buffy coat were nonspecific. For example, in patients with COVID-19, the lack of segmentation of neutrophil polymorphonuclear cells, the presence of apoptotic cells etc. are similar to those reported in the literature [21,22,23,24,25]. However, these changes are not specific and have been reported to be the consequence of acute diseases and/or complicated infectious diseases. The immunohistochemical study demonstrated granular cytoplasmic reactivity in platelets, lymphocytes and monocytes for antibodies directed against the nucleoprotein and the spike protein, both when SARS-CoV-2 RNA was expressed and when it was not. One explanation for this immunoreactivity may be the presence of viral remains on the surface of platelets, lymphocytes and monocytes or the presence of viral elements that have been phagocytosed by phagocytic mononuclear cells.

Our study has some limitations: (a) The small sample size and the high proportion of individuals with chronic comorbidities render it impossible to appreciate a clear association between viremia and severe COVID-19. Furthermore, most of our patients required admission to the intensive care unit, which is related to a worse prognosis. (b) The plasma RNA concentration and the viral RNA were determined on one occasion only; therefore, no information was obtained on the status of viral sepsis. (c) The infectivity of the SARS-CoV-2 found in both the plasma and the buffy coat could not be evaluated. Therefore, we could not determine whether the RNA detected is an intact infectious virus or an inactive nucleic acid that has no replicative capacity.

In conclusion, in the present study, we demonstrated that the plasma RNA concentration in patients with COVID-19 arises from viral RNA and that the presence of a gene component is related to a worse prognosis. The role of the buffy coat in COVID-19 should be investigated to determine if it is a limited source of the virus that is detected in plasma. The presence of viral components in platelets and monocytes may explain the capacity for complications in some groups of patients.

## 4. Materials and Methods

### 4.1. Study Design and Patient Cohort

This is a prospective and descriptive cohort study conducted on patients infected with SARS-CoV-2 admitted to the Sant Joan University Hospital, Reus, Spain, between December 2020 and March 2021. The study protocol was reviewed and approved by the hospital’s ethics committee of the Sant Joan University Hospital in Reus (registration number CEIM: 081/2020). Written informed consent was obtained from each subject in accordance with the principles of the Declaration of Helsinki 1964 and its subsequent modifications. We studied 24 consecutive blood samples from patients who were positive for SARS-CoV-2 infection. The general criteria for patient selection were local symptoms in the upper respiratory tract or with nonspecific symptoms, such as fever or muscle pain; positive test for SARS-CoV-2 infection by reverse transcription polymerase chain reaction (RT-PCR), using swab samples from the upper respiratory tract (nasopharyngeal swab); and SARS-CoV-2 pneumonia confirmed by chest X-ray and an ambient oxygen saturation of less than 90%. This study collected punctual blood specimens from COVID-19 patients hospitalized with a disease course of 1–91 days. The control group consisted of eight liquid biopsies from patients with lung carcinoma between December 2020 and March 2021. Before obtaining the liquid biopsy, a SARS-CoV-2 RT-PCR nasopharyngeal swab was performed, which was required to be negative to be included in the study. All of the patients were analyzed in the molecular pathology unit of our pathology department.

### 4.2. Blood Samples

**Scheme 1:**
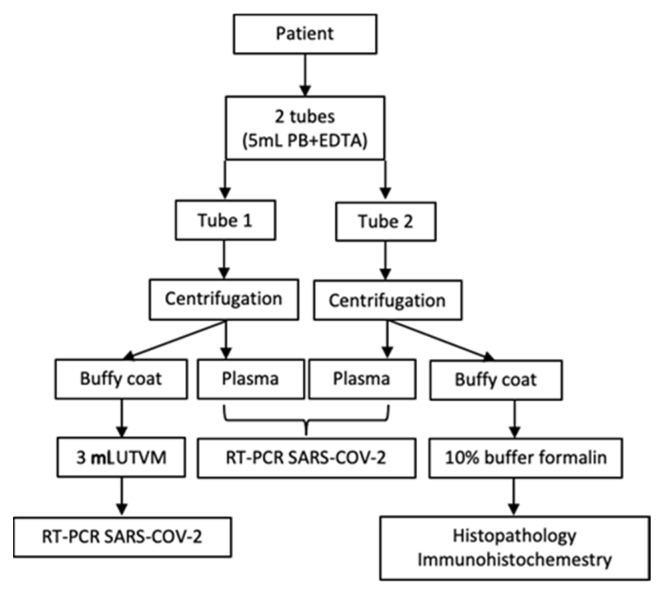
Schematic summary of the processing of the evaluated samples. PB: peripheral blood.

Two tubes with 5 mL of peripheral blood with EDTA were obtained from each patient and processed according to the following protocol (Scheme 1). The tubes were centrifuged at 1600× *g* for 10 min. Subsequently, the plasma was transferred to RNase-free Eppendorf tubes and immediately frozen at −80 °C. Once the plasma had been extracted, a second centrifugation of the remaining material was carried out at 1600× *g* for 10 min. Once the above process had been carried out, the buffy coat was gently aspirated, placed in 3 mL of universal viral transport medium, and immediately homogenized and frozen at −80 °C.

### 4.3. RNA Plasma Extraction

RNA was extracted from plasma samples with the High Pure Viral RNA Large Volume Kit (Roche) following the manufacturer’s instructions. Briefly, 500 uL of plasma samples were mixed with a working solution containing proteinase K, poly (A) and binding buffer. After incubation at 70 °C, samples were placed in a 50 mL tube with a spin column and centrifuged for 5 min at 4000 rpm. Then, the column was placed in an elution tube, and samples were subjected to several washing steps. Finally, RNA was eluted in 50 mL of elution buffer.

A curve for each of the studied genes was developed from a control with a known viral load. The ORF gene curve was chosen to calculate the plasma viral load.

### 4.4. RNA Buffy Coat Extraction

RNA was obtained from the buffy coat with universal viral transport media using the MagMAX Viral/Pathogen II Nucleic Acid Isolation Kit (Thermo Fisher Scientific, Waltham, MA, USA) and an automated KingFisher Flex System (Thermo Fisher Scientific). Briefly, 200 uL of each sample was dispensed into a deep-well plate. Then, 265 uL of binding solution was dispensed into each well, and the plate was incubated at room temperature for between 15 and 45 min. Then, 5 uL of proteinase K and 10 uL of magnetic beads were dispensed into each well. Finally, the deep-well plate with the samples as well as the deep-well plates with 80% ethanol, wash buffer and elution buffer were loaded onto the KingFisher Flex System to proceed with the automated extraction.

In both extraction procedures, the internal control for the PCR reaction (MS2) was added. RNA quantification was performed with a NanoDrop spectrophotometer (Thermo Fisher Scientific). To do so, 2 uL of each sample was used to quantify the RNA concentration. Measurements were performed in duplicate.

The kit used for the PCR reaction was the TaqPath COVID-19 CE-IVD RT-PCR (Life Technologies). The mastermix was prepared following the manufacturer’s instructions. Then, 15 uL of the mastermix and 10 uL of samples and controls (positive control and negative control) were loaded onto a microplate. The reaction was conducted in a QuantStudio 5 (Applied Biosystems, Waltham, MA, USA), and the results were interpreted using the *COVID-19 Interpretive Software* (Thermo Fisher Scientific).

### 4.5. Histopathological Preparation of the Buffy Coat

After plasma had been extracted, the buffy coat was fixed with 10% buffered formalin in the same tube at room temperature for at least 24 h. Subsequently, the buffy coat was removed and processed for conventional histopathological study. For each patient, 2 µm sections of paraffin-embedded material were obtained and stained with hematoxylin-eosin and Giemsa. Each histopathological study evaluated the presence of cells in the buffy coat, such as platelets, monocytes and lymphocytes, and studied whether morphological alterations could be found in the cellular components analyzed.

### 4.6. Immunohistochemistry Study of the Buffy Coat

All the buffy coat samples that were studied histopathologically were immunohistochemically stained and placed in a VENTANA^®^ Benchmark ULTRA/LT automatic immunohistochemistry processor (Ventana Medical Systems, Oro Valley, AZ, USA). The standardized protocol for SARS-CoV-2 detection was used: a recovery solution of pH 9 for 40 min at 100 °C and the Optiview^®^ DAB Immunohistochemistry Detection Kit (VENTANA^®^). The primary anti-SARS-CoV-2 NP antibody, clone 6F10 (BioVision Incorporated^®^, Milpitas, CA, USA), and the SARS-CoV-2 spike protein S2 antibody, MA5-35946 (Invitrogen^®^, Carlsbad, CA, USA), were reconstituted with 100 mL of distilled water at a dilution of 1:1000 and incubated for 32 min at 36 °C. Finally, the histological slides were developed with diaminobenzidine, contrasted with Mayer’s hematoxylin, dehydrated with alcohols at increasing concentrations, rinsed with xylol, and finally examined under an Olympus BX41 light microscope with direct increases ranging between 3.5× and 60×. SARS-CoV-2 positivity was considered when cytoplasmic granular labeling was obtained in cells from the buffy coat samples. Positive external controls for each antibody were used. RT-PCR SARS-CoV-2 assay results were unknown at the time that the immunohistochemical studies were evaluated.

### 4.7. Statistical Analysis

The results of the cellular analysis are shown as means ± SD and percentages. The differences in the results of the cell count and SARS-CoV-2 test were examined by the non-parametric Mann–Whitney test for two independent groups, with *p* < 0.05 being considered statistically significant. All of the analyses were performed using IBM.SPSS version 23.

## Figures and Tables

**Table 1:** Clinical characteristics of COVID-19 patients (* N = 24).

Patient	N	%
Male	18	75
Female	6	25
**Symptoms**	22	91.67
Dry cough	21	95.45
Fever	21	95.45
Dyspnea	20	90.91
Odynophagia	15	68.18
Anosmia	6	68.18
GI symptoms	1	4.55
**Asymptomatic**	2	8.33
**Intensive care unit**		
Required	13	54.17
Not required	11	45.83
**Lived**	16	66.67
**Died**	8	33.33

* N: Total number patients.

**Table 2:** Relevant clinical findings in patients with SARS-CoV-2 infection and the control group.

Patient	Gender	Age (Years)	Comorbidities	Symptoms	SARS-CoV-2 **	Initial O2 Sat. (%)	C-Reactive Protein (mg/dL)	X-ray Diagnostic (Lung)	Evolution
1	Male	69	DLP, CRCa	Cough, Fever, Dyspnea	Positive	91	12.78	Bilateral basal condensation	Live
2	Female	77	SAH	Cough, Fever, Dyspnea	Positive	95	22.2	Left basal condensation	Died
3	Male	51	Hyperglycemia	Fever, Dyspnea	Positive	94	17.6	Bilateral basal condensation	Live
4	Male	54	SAH, T2DM, DLP	Cough, Fever	Positive	95	15.7	Bilateral alveolar infiltrates	Live
5	Female	58	BRCa	Cough, Fever, Dyspnea	Positive	90	30	Bilateral peripheral infiltrates	Live
6	Female	54	Hypothyroidism	Cough, Fever, Dyspnea	Positive	87	14.72	Bilateral interstitial infiltrates	Live
7	Male	72	SAH, T2DM, DLP	Cough, Dyspnea	Positive	95	28	Bilateral interstitial infiltrates	Died
8	Male	78	SAH	Cough, Fever, Dyspnea	Positive	93	4.85	Bilateral interstitial infiltrates	Died
9	Male	63	T2DM	Fever, Dyspnea	Positive	70	11	Bilateral basal condensation	Live
10	Female	73	SAH, DLP	Cough, Fever, Dyspnea	Positive	98	NR	Bilateral interstitial infiltrates	Live
11	Male	62	SAH, T2DM, DLP	Fever, Dyspnea	Positive	85	22	Bilateral interstitial infiltrates	Died
12	Male	62	SAH, T2DM, CRI	Cough, Fever, Dyspnea	Positive	90	18.7	Bilateral interstitial infiltrates	Died
13	Male	57	SAH, DLP	Cough, Dyspnea	Positive	65.8	13	Bilateral interstitial infiltrates	Live
14	Male	69	SAH, DLP	Cough, Dyspnea	Positive	65	16	Bilateral basal condensation	Live
15	Male	61	No	Cough, Fever, Dyspnea	Positive	75	25	Bilateral basal condensation	Live
16	Male	54	DLP	Cough, Fever, Dyspnea	Positive	92	6.31	Bilateral interstitial infiltrates	Live
17	Male	69	No	Cough, Fever, Dyspnea	Positive	90	14.3	Bilateral basal condensation	Live
18	Female	76	CRI	Diarrhea	Positive	97	10	Right basal condensation	Died
19	Male	82	SAH, DLP	Cough, Dyspnea	Positive	88	4.2	Bilateral interstitial infiltrates	Died
20	Female	63	SAH, T2DM, DLP	Cough, Fever, Dyspnea	Positive	90	16.93	Bilateral basal condensation	Live
21	Male	53	SAH, DLP	Asymptomatic	Positive	98	NR	No infiltrates. No consolidations	Live
22	Male	54	SAH, Obesity	Asymptomatic	Positive	98	NR	No infiltrates. No consolidations	Live
23	Male	51	DLP	Cough, Fever, Dyspnea	Positive	94	13	Bilateral interstitial infiltrates	Live
24	Male	77	SHA, DLP	Cough, Fever, Dyspnea	Positive	88	6.14	Bilateral basal condensation	Died
**Control**									
1	Male	72	CRCa	Asymptomatic	Negative	-	-	-	Live
2	Male	52	LCa	Asymptomatic	Negative	-	-	-	Live
3	Male	51	LCa	Asymptomatic	Negative	-	-	-	Live
4	Female	62	CRCa	Asymptomatic	Negative	-	-	-	Live
5	Female	62	LCa	Asymptomatic	Negative	-	-	-	Live
6	Male	75	CRCa	Asymptomatic	Negative	-	-	-	Live
7	Female	64	CRCa	Asymptomatic	Negative	-	-	-	Live
8	Female	68	LCa	Asymptomatic	Negative	-	-	-	Live

** RT-PCR from a nasopharyngeal swab. Abbreviations: DLP: dyslipidemia. SAH: systemic arterial hypertension. T2DM: type 2 diabetes mellitus. BRCa: breast carcinoma. CRCa: colorectal carcinoma. LCa: lung carcinoma. CRI: chronic renal insufficiency. NR: not referred. Sat.: Saturation.

**Table 3:** Patients with RT-PCR SARS-CoV-2 findings in both plasma and the buffy coat.

Case	Age	Sex	Gene Plasma	Gene Buffy Coat	Evolution
2	77	F	ORF, S, N	ORF, N	Died
4	54	M	N	N	Live
5	58	F	ORF, S	Negative	Live
6	54	F	ORF, N, S	N, S	Live
7	72	M	ORF	Negative	Died
8	78	M	ORF, S	Negative	Died
9	63	M	ORF, S	Negative	Live
13	57	M	ORF, S	Negative	Live
14	69	M	ORF, S	Negative	Live
17	69	M	ORF, N, S	ORF, N	Live
18	76	F	ORF	Negative	Died
19	82	M	ORF, S	Negative	Live
23	77	M	ORF, S	Negative	Died

F: female; M: male. ORF: Open Read Frame gene. N: Nucleocapsid gene. S: Spike gene.

## Data Availability

All of the data are present in the manuscript.
